# Safety of on-scene medical care by EMS nurses in non-transported patients: a prospective, observational study

**DOI:** 10.1186/s13049-018-0540-z

**Published:** 2018-09-14

**Authors:** Wim Breeman, Nathan A. Poublon, Michael H. J. Verhofstad, Esther M. M. Van Lieshout

**Affiliations:** 1 AmbulanceZorg Rotterdam-Rijnmond, P.O. Box 4, 2990 AA Barendrecht, The Netherlands; 2 Trauma Research Unit Department of Surgery, Erasmus MC, University Medical Center Rotterdam, P.O. Box 2040, 3000 CA Rotterdam, The Netherlands

**Keywords:** EMS, Ambulance, Non-transport, Non-conveyance, Paramedics, On-scene, Care on the scene

## Abstract

**Background:**

After on-scene examination and /or treatment, emergency medical services (EMS) nurses must decide whether the patient requires further assessment or treatment, most frequently in a hospital. The primary objective of this study was to assess the reliability of the current EMS protocol by determining whether the decision not to transport the patient to a care provider was correct or not.

**Methods:**

Adults receiving on-scene medical care by an EMS rapid responder or full team without transport to the hospital were included in this prospective observational study. The primary outcome measure was secondary consultation within 24 h after an on-scene EMS evaluation without transport for the same or a closely related complaint. The secondary outcome measures were patient satisfaction, type of secondarily consulted health care provider, provisional and definitive diagnosis, and correctness of the EMS members’ decision to provide on-scene medical care without transport.

**Results:**

Of the 1095 participating patients, 271 (24.7%) patients requested secondary medical attention for the same complaint. This percentage was significantly larger in incidents attended by an ambulance team than by a rapid responder (*N* = 248 (26.5%) vs. *N* = 23 (14.4%); *p* < 0.05). In eleven (1.0%) cases an urgent medical diagnosis requiring admission was missed. A total of 873 (79.7%) patients were satisfied with the decision not to be transported. In 44 (4.0%) cases the EMS nurse’s decision was rated incorrect since the patient needed help contradictory to the EMS nurse’s recommendation.

**Conclusions:**

The data show that EMS nurses can effectively examine patients, but a low threshold of referral for consultation should be considered because one in four patients requested secondary medical attention for the same complaint(s) again. However, due to a low response rate (11.3%) more research is needed to further determine the safety of the current EMS protocol.

**Trial registration:**

Not applicable.

## Background

In the Netherlands, patients may seek acute medical attention by contacting their own general practitioner (GP), a GP employed at an out-of-hours GP services unit, a clinical emergency department (ED) or a regional EMS Dispatch Center. Based upon the type of call, an ambulance or rapid responder unit is dispatched. EMS nurses in the Netherlands are fully registered nurses with additional certification in Intensive Care, ED, or anesthesia nursing before they are allowed to apply for an EMS nurse educational program. In addition to ‘on-the-job-training’ they have to complete a nine-month ambulance educational program during which they are supervised by an experienced EMS teaching nurse. After successful completion of the training, EMS nurses are legally authorized to carry out medical procedures according to the nationwide ambulance protocols based on provisional diagnoses from clinical signs, symptoms and mobile point-of-care diagnostic tools. Traditionally, the EMS interventions mean to be resuscitative until a final diagnosis and definitive care is provided by a physician in, e.g., a hospital. EMS nurses operate in an ambulance team with a driver (without a nursing or medical degree) or as a solitary rapid responder in a car or on a motorbike without the possibility to transport a patient.

However, the number of on-scene medical care without transport dispatches is gradually increasing, both within and outside The Netherlands, due to a variety of reasons, e.g., population growth, a tendency of patients to activate EMS in non-life-threatening situations, and failing triage systems [1]. In The Netherlands, on-scene medical care without transport is applied for a wide range of medical complaints. A pilot study in our region showed that ambulance teams and rapid responders do not transport patients in 14.7% and 66.3% of cases, respectively [2]. Two small retrospective studies suggest that a decision not to transport a patient to a hospital was justified in 93–99% of cases [3, 4], but data on the outcomes of such patients are not readily available in the (inter)national literature [5].

Internationally, several studies indicate that paramedics can safely decide to leave patients at home [6, 7], while other research indicates that if the non-conveyance rate increases, patient safety could be compromised [8, 9]. Thus, results of these studies are inconclusive. Moreover, the EMS system and the level of education of health care providers in the countries where these studies have been conducted, differ from The Netherlands and results cannot be easily extrapolated.

The primary objective of the current study was to determine the percentage of patients who received secondary medical assistance for, or died from the same complaint, within 24 h after the primary call to the regional EMS dispatch center and delivered care without transport. Secondary aims were to analyze (1) patient satisfaction after a non-transport decision, (2) whether patients subsequently consulted another health care provider, (3) differences between the provisional diagnosis made between the EMS nurse and the final diagnosis by the consulting physician and (4) to investigate the correctness of the EMS nurse’s decision to provide on-scene medical care without transport, comparing all results between rapid responders and ambulance teams.

## Methods

### Study design

This study followed a prospective observational study design and was conducted from April 8, 2014 until February 25, 2015.

### Study population

The region Rotterdam-Rijnmond has 1.3 million inhabitants. The EMS dispatch center receives 126,000 requests in this region for help annually. Ambulancezorg Rotterdam Rijnmond (AZRR) is the only EMS provider and services all inhabitants. AZRR has 60 advanced life support (ALS) ambulances, seven rapid responder units, four motorbikes, six low-care ambulances for interhospital transport, and one nurse practitioner/supervisor unit.

All patients aged 18 years or older who were evaluated on-scene by EMS but not transported to the hospital were eligible for inclusion, regardless of weather conditions. There was no split-up in groups based on patient characteristics. Based on the nationwide ambulance protocols, all high-risk and unstable patients were transported to the hospital. Each participating patient signed written informed consent. Patients who were deceased on arrival of the EMS nurse, patients without a permanent address, and patients with insufficient comprehension of the Dutch language were excluded. When the EMS nurse forgot to ask the patient for participation, the patient was excluded based on the absence of informed consent.

### Outcome measures and data collection

The primary outcome measure was secondary medical consultation or death due to the same medical complaint. The timeframe for this consultation was set to 24 h after on-scene medical care without transport to be sure both were requested for the same complaint.

Secondary outcome measures were patient satisfaction, type of secondarily consulted health care provider, provisional and definitive diagnosis, and the correctness of the EMS nurses’ decision to give on-scene medical care without transport.

Baseline characteristics of each patient were taken from the EMS nurse’s reporting on the non-transportation-associated form. A research assistant called each patient within 10 days after the on-scene medical care attendance. A partially multiple-choice interview was conducted. Patients were asked about secondary care, follow-up treatment and satisfaction about care provided by the EMS team. Patients who sought secondary care were asked for written permission to contact the consulted physician.

A research assistant contacted the physician and asked for information about the time and date of the consultation, the definitive diagnosis, if the complaint was related to the first complaint, referral of the patient, and agreement with the non-transportation policy. Correctness of the EMS nurse’s decision to non-transport the patient was assessed by comparing the recommendation of the EMS nurse on the non-transportation form with the presence of a secondary request for help from the patient.

### Procedures

The EMS dispatcher responsible for the intake of emergency calls is always a certified nurse with additional dispatcher training. Based on the dispatcher’s triage an ambulance or EMS unit with a rapid responder is sent to the incident site. Upon arriving on the scene, the EMS nurse examines the patient and generates a provisional diagnosis. Subsequently, treatment is initiated as applicable. If definitive care can be provided at the scene, the patient is not transported to an ED in the current protocol [10]. This is the decision of the EMS nurse. Each patient receives verbal information and a written statement about this decision and his GP is notified.

Non-transported patients received verbal and written explanation on the study. After written permission, they were called by telephone within the next seven days to ask for participation. If the patient had contacted an EMS, GP, or hospital within 24 h after receiving on scene EMS evaluation, they were asked for permission to contact that EMS, GP or hospital by signing informed consent. Finally, after written permission of the patient, the additional information required for this study was obtained from these health care providers.

### Sample size

Sample size calculation was based on findings by Schmidt et al., reporting that the decision not to bring patients to a hospital is incorrect in 9.3% of cases [11]. To reliably identify 100 unjustly non-transported patients, a total of 1100 non-transportation dispatches needed to be included in this study.

### Statistical analysis

Data were analyzed using the Statistical Package for the Social Sciences (SPSS) version 21 (IBM Corp. Released 2012. IBM SPSS Statistics for Windows, Version 21.0. Armonk, NY: IBM Corp.). The normality of continuous data was tested with the Shapiro-Wilk test, and the homogeneity of variance was tested using Levene’s test. Continuous data were all non-normally distributed. Descriptive statistics were performed to report EMS attendance and patient characteristics. For continuous data medians and percentiles were calculated. For categorical data frequencies were calculated. The percentage of patients who sought secondary medical attention within 24 h after non-transportation was calculated and the statistical significance of the mean difference in this percentage between the rapid responder and the ambulance team was tested with the chi-squared test. The difference in secondary outcome measures between rapid responder and ambulance teams was tested using the Mann-Whitney U-test (continuous data) or the chi-squared test (categorical data). A *p*-value < 0.05 was taken as the threshold of statistical significance.

### Ethics approval

The study was exempted by the local Medical Research Ethics Committee. All study patients provided written informed consent.

## Results

**Fig. 1:**
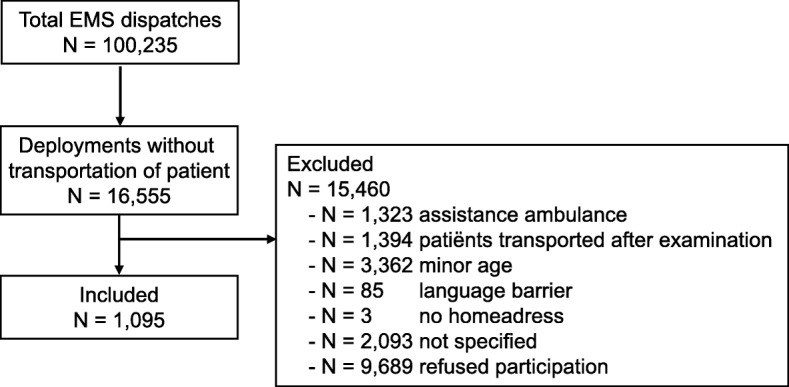
Study flow chart

During the research period, 100,235 ambulance team and rapid responder dispatches were carried out by the EMS, of which 16,555 (16.5%) involved on-scene medical care without transport of the patient (Fig. 1). A total of 8260 (46.0%) dispatches were excluded based on the exclusion criteria and 9689 patients refused to participate. A total of 1095 unique patients (11.3%) consented to participate.

The median age in the study population was 47 years (P_25_-P_75_ 30–64) and 480 patients (43.8%) were male (Table 1). The non-transportation dispatches were predominantly carried out by an ambulance team (*N* = 935; 85.4%). Panic disorders such as hyperventilation were the most frequent reasons for EMS activation (*N* = 209; 19.1% of calls), followed by small traumatic injuries (*N* = 197; 18.0%), and collapse of unknown cause with vasovagal syncope (*N* = 145; 12.9%). The remaining cases reflected a wide range of problems, often without an evident medical diagnosis (*N* = 107; 9.8%). Patients of the EMS dispatches carried out by a rapid responder were 8.5 years younger on average and more often female than the dispatches carried out by a full ambulance team. In dispatches carried out by a rapid responder, burns, epistaxis, and small injuries were more frequent.

**Table 1:** Baseline characteristics of EMS dispatches for the total population and separated by type of EMS care provider

Variable	Overall population (*n* = 1095)	Ambulance team (*n* = 935; 85.4%)	Rapid responder (*n* = 160; 14.6%)
Age	47 (30–64)	48 (31-63)	45 (28-67)
Male	480 (43.8%)	426 (45.6%)	54 (33.8)
Provisional diagnosis			
Non-specific abdominal pain	38 (3.5%)	34 (3.6%)	4 (2.5%)
Angina pectoris	15 (1.4%)	14 (1.5%)	1 (0.6%)
Non-specific thoracic pain	19 (1.7%)	18 (1.9%)	1 (0.6%)
Burns	4 (0.4%)	1 (0.1%)	3 (1.9%)
Collapse	95 (8.7%)	80 (8.6%)	15 (9.4%)
Dizziness	18 (1.6%)	15 (1.6%)	3 (1.9%)
Epilepsy	34 (3.1%)	32 (3.4%)	2 (1.3%)
Epistaxis	7 (0.6%)	5 (0.5%)	2 (1.3%)
Head/neck injury	38 (3.5%)	34 (3.6%)	4 (2.5%)
Hypoglycemia	51 (4.7%)	45 (4.8%)	6 (3.8%)
Infection	37 (3.4%)	32 (3.4%)	5 (3.1%)
Intoxication	26 (2.4%)	23 (2.5%)	3 (1.9%)
Small traumatic injuries	197 (18.0%)	153 (16.4%)	44 (27.5%)
Colic	7 (0.6%)	7 (0.7%)	NA
Myalgia	46 (4.2%)	38 (4.1%)	8 (5.0%)
Unknown	107 (9.8%)	100 (10.7%)	7 (4.4%)
Other	63 (5.8%)	51 (5.5%)	12 (7.5%)
Palpitations	9 (0.8%)	9 (1.0%)	NA
Panic attack	209 (19.1%)	180 (19.3%)	29 (18.1%)
Arrhythmia	11 (1.0%)	10 (1.1%)	1 (0.6%)
Socio-psychiatric complaints	15 (1.4%)	12 (1.3%)	3 (1.9%)
Vasovagal syncope	49 (4.5%)	42 (4.5%)	7 (4.4%)

Data are shown as median (P_25_-P_75_) or as number (%)

Table 2 shows that in 634 (57.9%) non-transportation dispatches all completed forms were present upon return to the ambulance station. On 369 (56.3%) of the forms the EMS nurse recommended that the patient visit a physician after non-transportation.

**Table 2:** Outcome of EMS dispatches after non-transportation

Variable	Overall population (*n* = 1095)
Completeness of the administrative reporting	
Complete	634 (57.9%)
No reporting	21 (1.9%)
No non-transportation form	337 (30.8%)
Both incomplete	103 (9.4%)
Recommendation to seek secondary medical care	
Yes	369 (33.7%)
No	283 (25.8%)
Unknown	443 (40.5%)
Information provider	
Patient	818 (74.7%)
Alternative contact	105 (9.6%)
Next of kin	3 (0.3%)
Non-responding	169 (15.4%)
Patient satisfaction	
Satisfied	873 (79.7%)
Indifferent	14 (1.3%)
Unsatisfied	36 (3.3%)
Would not tell/unknown	172 (15.7%)
Secondary medical attention requested	
No	651 (59.5%)
Yes	271 (24.7%)
Would not tell/unknown	173 (15.8%)
Secondary medical attention provided	
No	717 (65.5%)
Yes	205 (18.7%)
EMS	1 (0.1%)
GP	118 (10.8%)
Out of Office Hours GP services unit	35 (3.2%)
Emergency Department	48 (4.4%)
Unknown	173 (15.8%)

Data are shown as Number (%)

Non-transportation, on-scene medical care without transport; *EMS* emergency medical services, *GP* general practitioner

An interview was conducted in 926 (84.6%) of the included dispatches and in 818 (88.3%) of these cases the patient was contacted directly. A total of 105 (11.3%) alternative contacts were interviewed due to repetitive unavailability of the patient at the time the research assistant called, and three (0.3%) relatives were interviewed because the patient was deceased. Among these three cases, two patients appeared to be in a terminal stage of a chronic condition and in the other case the family was not able to provide any relevant information. In one case the patient died due to a traffic accident, unrelated to the initial request for help, before he could be contacted at all. No deaths were related to non-transportation. Four (0.4%) patients were unwilling to answer the research assistant’s questions and withdrew consent to participate in the study.

Eight-hundred-seventy-three (94.7%) interviewed persons reported to be satisfied about the medical assistance (Table 2).

Two-hundred-seventy-one (29.4%) patients reported having requested secondary medical attention after non-transportation. In 118 (57.6%) cases the patient’s own GP provided secondary medical attention, followed by 48 (21.5%) patients visiting the ED of the hospital and 35 (17.1%) visiting an out-of-hours GP services unit. One patient secondarily requested medical help with his own GP who activated the EMS and the patient was transported to the hospital.

In 319 (29.1%) of the dispatches the patients followed the recommendation of the EMS nurse to either seek secondary help or to refrain from this request (Table 3). In 204 (18.6%) dispatches the patient did not consult a physician despite the EMS nurse’s recommendation. In 44 (4.0%) of the dispatches the patient did consult a physician despite the discouragement of the EMS nurse. In the other 528 (48.2%) cases a recommendation and/or a non-transportation-associated form was absent and the correctness of the EMS nurses’ decision remains unknown.

**Table 3:** Correctness of EMS provider

Correctness of the decision to not-to transport	Overall population (*n* = 1095)
No request for medical attention conforming with recommendation	205 (18.7%)
No request for medical attention despite recommendation	204 (18.6%)
Request for medical attention conforming with recommendation	114 (10.4%)
Request for medical attention despite recommendation	44 (4.0%)
Unknown	528 (48.2%)

Data are shown as N (%)

Table 4 shows that rapid responders were 14.9% more likely to fully complete the forms (*N* = 113 versus 521) and 6.7% more likely to advise the patient to visit a physician (*N* = 63 versus 306) compared with an ambulance team. Both differences reached statistical significance. No significant difference in patient satisfaction regarding the EMS nurses’ decision not to transport the patient between rapid responder and ambulance team was found. Compared with patients seen by a rapid responder, patients who were left on site by an ambulance team both requested (*N* = 23 versus 248; 12.1%) and received (*N* = 18 versus 187; 8.7%) secondary care more frequently. No significant difference in distribution between secondary health care providers was found.

**Table 4:** Differences in outcome after non-transportation between ambulance team and rapid responder

Variable	Ambulance team (*n* = 935)	Rapid responder (*n* = 160)	*P*-value
Completeness of the nurse’s reporting			
Complete	521 (55.7%)	113 (70.6%)	**0.001**
No reporting	17 (1%)	4 (2.5%)	
No non-transportation associated form	310 (33.2%)	27 (16.9%)	
Both incomplete	87 (9.3%)	16 (10.0%)	
Recommendation to seek secondary medical care			
Yes	306 (32.7%)	63 (39.4%)	**0.005**
No	232 (24.8%)	51 (31.9%)	
Unknown	397 (42.5%)	46 (28.7%)	
Patient satisfaction			
Satisfied	751 (80.3%)	122 (76.3%)	0.313
Indifferent	12 (1.3%)	2 (1.3%)	
Unsatisfied	27 (2.9%)	9 (5.6%)	
Would not tell/unknown	145 (15.5%)	27 (16.9%)	
Secondary medical attention requested			
No	541 (57.9%)	115 (71.9%)	**0.004**
Yes	248 (26.5%)	23 (14.4%)	
Unknown	146 (15.6%)	27 (16.9%)	
Secondary medical attention provided			
No	602 (64.4%)	115 (71.9%)	**0.018**
Yes	187 (20.0%)	18 (11.3%)	
EMS	1 (0.3%)	NA	0.418
GP	108 (32.4%)	10 (22.2%)	
Out of Office Hours GP services unit	32 (9.6%)	3 (6.7%)	
Emergency Department	43 (12.9%)	5 (11.1%)	
Unknown	146 (15.6%)	27 (16.9%)	

Data are shown as N (%)

*EMS* emergency medical services, *GP* general practitioner

Boldface in the Table indicates significance

Patients seen by an ambulance team did not follow up the recommendation of the EMS nurse significantly more often than patients seen by a rapid responder (Table 5).

**Table 5:** Differences in correctness separated by EMS provider

Variable	Ambulance team (*n* = 935)	Rapid responder (*n* = 160)	*P*-value
Correctness of the decision not to transport			
No request for medical attention conforming with recommendation	163 (17.4%)	42 (26.3%)	**0.005**
No request for medical attention despite recommendation	166 (17.8%)	38 (23.8%)	
Request for medical attention conforming with recommendation	100 (10.7%)	14 (8.8%)	
Request for medical attention despite recommendation	41 (4.4%)	3 (1.9%)	
Unknown	465 (49.8%)	63 (39.5%)	

Data are shown as number (%)

Boldface in the Table indicates significance

In this case, patients were 2.5% (*N* = 3 versus 41) more likely to request secondary medical attention despite the EMS nurse’s advice that this was not indicated.

Table 6 shows that patients who consulted a physician were predominantly seen within one day after non-transportation. Of these patients, 163 (79.5%) signed informed consent allowing the researchers to contact their physician regarding the care that had been provided during the EMS assessment. The research assistant spoke to the patient’s GP in 92 (74.2%) of the cases. In only 39 (19.0%) of the cases was the physician not reached or did not give consent to be interviewed. The research assistant spoke to 19 (15.3%) GPs employed at an out-of-hours GP services unit and to eight (10.5%) physicians who worked at the ED of the hospital. Small traumatic injuries were seen most frequently (*N* = 25, 12.2%) in the secondary consults, followed by 18 panic attacks (8.8%), nine viral infections (4.4%), six myalgia attacks (2.9%), and five nonspecific thoracic pain attacks (2.4%). In none of these cases was emergency medical care indicated. No medical problem was found in 21 cases (10.2%), and the diagnosis remained unclear in one (0.5%) case. The remaining cases reflected a wide variety of medical problems (*N* = 38; 18.5%).

**Table 6:** Characteristics of re-assessment by a physician after non-transportation dispatches

Variable	Overall populatinpopulation (*n* = 205)
Delay of re-assessment by a physician, hours	12.6 (12.9%)
Complaint deteriorated at the time of request	
Yes	43 (22.9%)
No	86 (54.6%)
Won’t tell/unknown	46 (22.4%)
Consent to contact physician	
Yes	163 (79.5%)
No	42 (20.5%)
Health care provider	
General Practitioner	92 (44.9%)
Out of Office Hours GP services unit	19 (9.3%)
Emergency Department	13 (6.3%)
Unknown/Not applicable	81 (39.5%)
Definitive diagnosis	
Adverse effect medication	2 (1.0%)
Arrhythmia	2 (1.0%)
Asthma exacerbation	1 (0.5%)
Collapse	2 (1.0%)
Concussion	2 (1.0%)
Cystitis	1 (0.5%)
Fracture	3 (1.5%)
Insult	4 (2.0%)
Intestinal bleeding	1 (0.5%)
Myalgia (no trauma)	6 (2.9%)
Nephrolithiasis/Cholecystolithiasis	4 (2.0%)
No consult	5 (2.4%)
No pathology	16 (7.8%)
Non-specific thoracic pain	5 (2.4%)
Nnon-ST-elevation myocardial infarction	1 (0.5%)
Other medical	7 (3.4%)
Pancreatitis	2 (1.0%)
Panic attack	18 (8.8%)
Pneumonia	3 (1.5%)
Small traumatic injuries	25 (12.2%)
Transient Ischemic Attack	3 (1.5%)
Unknown	82 (40.0%)
Vasovagal syncope	4 (2.0%)
Viral infection	6 (2.9%)

Data are shown as N (%)

In 30 (14.6%) interviews, the physician reported having referred the patient to a tertiary health care provider (Table 7). Eleven (5.5%) cases consisted of a more serious problem where urgent admission to hospital was indicated such as cystitis, pneumonia, cholecystolithiasis, pancreatitis, intestinal bleeding, and non-ST- segment-elevation myocardial infarction (NSTEMI). None of the physicians reported that the patient died as a consequence. The secondarily consulted physicians reported disagreeing with the EMS nurse’s decision not to transport the patient in 7 (3.4%) of the included EMS dispatches. There was no specific pattern or type of case the physician disagreed with.

**Table 7:** Follow-up of secondary consultations

Variable	Overall population (*n* = 205)
Referral	
Yes	30 (14.6%)
No	93 (45.4%)
Unknown/Not applicable	81 (28.7%)
Deceased	
Yes	NA
No	125 (61.0%)
Unknown	78 (39.0%)
Agreement of physician with decision not to transport	
Yes	102 (49.8%)
No	7 (3.4%)
Unknown	82 (40.0%)

Data are shown as N (%)

In only 0.4% (*N* = 4) of the investigated dispatches were provisional diagnoses found to be more severe after examination of a physician (e.g. contusion/sprain turning out to be a fracture, a common cold turning out to be a pneumonia, angina pectoris turning out to be a NSTEMI, and obstipation turning out to be pancreatitis).

The vast majority of patients reported being satisfied with the provided care and the decision not to transport them to a hospital. Only seven (3.4%) physicians disagreed with the correctness of the decision not to transport. However, this was after the diagnosis was confirmed in the clinic by diagnostic tests.

## Discussion

The primary objective of this study was to investigate the effectiveness and safety of current on-scene EMS evaluation without transport to a hospital. Approximately one in five patients received secondary medical attention for the same complaint within one day. The predominant share of these consults consisted of non-emergency medical care. Four percent of these patients requested secondary help despite the EMS nurses’ opinion this was not necessary. Only one in twenty secondary consultations revealed a more severe medical diagnosis than suggested by the EMS nurse. These low percentages show that it is relatively safe to choose not to transport a patient in the national ambulance-care protocol. Local adherence to a national protocol is mandatory for many reasons, especially for diagnoses with potentially dire consequences. Patients with clinical signs that could fit myocardial infarction should invariably be transported to an appropriate hospital, even in the absence of objective signs (e.g., because of the possibility of NSTEMI). On the other hand, transportation and presentation to a hospital of each minor trauma will inevitably result in a shortage of EMS capacity and overcrowded EDs. The balance between safety and efficacy is fragile and can be strengthened by technological improvements such as teleconsulting with telemonitoring in all EMS units.

Our results do fit into the perspectives that are observed in international studies. One paper, studying EMS care provided to children found that, depending on the medical complaint, 17–42% of the patients transported by ambulance could have been safely left on-site after on-scene assessment and treatment and that in 34% of the non-transportation dispatches no ambulance would have been needed at all [12]. Finding an alternative possibility to request help could increase the EMS dispatch response time and cost-effectiveness [13]. Regarding safety, several studies found that a non-transportation protocol decreases the workload and costs of EMS but endangers patient safety, since paramedics estimate correctly if a patient needs transport in 76% and ED-care in 55% of the dispatches, respectively [7, 14]. Sixty percent of the patients discharged by an EMS nurse had no complaints. Another study found that only 2.4% of the patients received secondary medical attention and hospital admission after non-transportation and a mean satisfaction of 4.4 on a five-point Likert scale regarding the decision not to transport the patient [9].

Non-surprisingly, the results showed that patients treated by an ambulance team without transportation received secondary medical attention more frequently than when treated by a rapid responder after non-transportation. Rapid responders are especially dispatched to patients with expected minor injuries or insignificant conditions since they do not have a transport possibility. However, in this study no superiority of either rapid responder teams or ambulance teams in outcome regarding the provided care or patient satisfaction was found.

### Limitations

A clear limitation of this study is the large amount of missing data. Only 11.3% of all eligible non-transportation patients provided informed consent. This may have compromised the internal validity of the findings. Several factors could have attributed to this low percentage. If a patient is in an emotional state it can be hard to find an appropriate moment to complete the required forms. Even so, weather conditions and working with limited time and pressure from the EMS dispatch center form another barrier completing administrative reporting. In a substantial number of dispatches, the EMS nurse simply forgot to ask for participation. This is a well-known phenomenon within the prehospital research setting; for most of these patients additional administrative reporting was also incomplete or not done at all [5, 14, 15]. A reporting bias might be present due to EMS nurses not including patients with a poorer prognosis. However, the baseline population characteristics found in this study are in line with the data from a previous retrospective file analysis [5]. Therefore, the assumption is made that the investigated population is representative of the population transported by the EMS services. Another important limitation is the number of patients who refused to cooperate (9689 patients) for various reasons, which could induce bias as well because the patients were not asked why they would not cooperate. A third limitation is the 15% loss to follow-up. Despite repetitive attempts to reach all included patients by email, mail, in person or by telephone, researchers did not succeed in interviewing them all. Another 20% of patients did not return a written consent to contact the consulted physician after they had been interviewed. So follow-up of secondary consultations is unknown, and whether outcomes differ between patients who cannot be contacted is still a subject of uncertainty. Recall bias is not very likely to play a significant part since all patients were contacted within one week after the non-transportation deployment, but it cannot be excluded totally, due to the design of this study.

## Conclusions

Current data show that EMS nurses can effectively examine the patient, initiate treatment when required, and make decisions about which patients do not need immediate transportation for further medical evaluation. The vast majority of non-transported patients was satisfied. Nevertheless, approximately one in four patients did seek additional medical consultation for the same complaint(s) within 24 h. This hardly yielded any additional diagnoses with therapeutic consequences. However, to draw firm conclusions with regard to safety and efficacy, additional research is required to address the main methodological flaw of the current study, which is a low response rate.

## Abbreviations

AZRR: Ambulancezorg Rotterdam Rijnmond
ED: Emergency department
EMS: Emergency medical services
GP: General practitioner
NSTEMI: Non-ST-segment elevation myocardial infarction
SPSS: Statistical Package for the Social Sciences
